# Mechanistic modelling of highly pathogenic avian influenza: A scoping review revealing critical gaps in cross-species transmission models

**DOI:** 10.1371/journal.pone.0347929

**Published:** 2026-04-30

**Authors:** Manting Wang, Elda K. E. Laison, Tanya Philippsen, Sajjad Ghaemi, Juxin Liu, Iain Moyles, Anthony Signore, Junling Ma, Bouchra Nasri

**Affiliations:** 1 Département de Médecine Sociale et Préventive, Université de Montréal, Montréal, Québec, Canada; 2 Department of Mathematics and Statistics, University of Victoria, Victoria, British Columbia, Canada; 3 Digital Technologies Research Centre, National Research Council Canada, Toronto, Ontario, Canada; 4 Department of Mathematics and Statistics, University of Saskatchewan, Saskatoon, Saskatchewan, Canada; 5 Department of Mathematics and Statistics, York University, Toronto, Ontario, Canada; 6 Canadian Food Inspection Agency (Western Area), Winnipeg, Manitoba, Canada; Universidad Cooperativa de Colombia, COLOMBIA

## Abstract

**Background:**

Highly pathogenic avian influenza (HPAI) viruses, particularly subtypes such as H5N1 and H7N9, have caused widespread outbreaks in wild birds, poultry, livestock and occasionally humans, raising concerns about cross-species transmission and pandemic potential. Effective control and surveillance strategies require a thorough understanding of HPAI transmission dynamics, which can be supported by mathematical modelling.

**Objective:**

This scoping review aimed to identify mechanistic models used to study HPAI transmission. Specifically, we sought to categorize model types, describe their application contexts (e.g., wild birds, poultry, livestock, and humans), and highlight modelling gaps relevant to understanding and mitigating the risks of HPAI spread.

**Methods:**

Following PRISMA guidelines and the PRISMA extension for scoping reviews (PRISMA-ScR), we conducted systematic searches of PubMed and Web of Science to identify peer-reviewed studies employing deterministic and stochastic models to analyze HPAI transmission. Eligible articles published between January 2023 and June 2025 were screened and grouped by model structure, host populations, transmission pathways, and modelling objectives.

**Results:**

After screening, 30 studies published after 2023 were included in this scoping review. Compartmental models were the most common (26 studies), with 16 deterministic and 10 stochastic approaches. These models were primarily used to describe transmission among wild birds, poultry, livestock, and humans and to evaluate interventions such as culling, vaccination, and movement restrictions. Agent-based models (2 studies) captured individual-level interactions and spatial heterogeneity, while network models (2 studies) represented contact structures and transmission pathways between farms or species.

**Conclusions:**

Currently, mechanistic modelling of HPAI is dominated by compartmental approaches, including both deterministic and stochastic formulations, whereas agent-based and network models remain relatively underused. Although most studies focus on transmission in wild birds and poultry, and in some cases spillover infections to humans, few explicitly examine infection dynamics in livestock or in transmission between livestock and humans, despite the importance of livestock (e.g., cattle) as potential intermediaries in human infection. Key gaps persist in the integration of empirical data, representation of multi-host interactions, and evaluation of realistic intervention strategies. Addressing these limitations is essential to improve predictive accuracy and to strengthen the role of modelling in informing HPAI surveillance and control.

## Introduction

### Rationale

The emergence and spread of avian influenza viruses (AIVs) threaten the health of poultry, livestock, wildlife, and humans [1–3]. Avian influenza A viruses are maintained in wild aquatic birds, which serve as the primary natural reservoir. Infected wild birds are typically asymptomatic or exhibit only mild symptoms, as they are usually infected with low pathogenic avian influenza (LPAI) viruses [4–6]. However, as wild birds migrate, they can transmit LPAI viruses to resident birds and domestic poultry. Notably, some LPAI subtypes, especially H5 and H7, have the potential to genetically mutate into highly pathogenic forms [7,8]. In contrast, highly pathogenic avian influenza (HPAI) viruses cause severe disease and high mortality in poultry, resulting in devastating economic losses and posing a significant threat to human safety [3,5].

The spread of HPAI viruses is facilitated by complex cross-species dynamics and diverse transmission pathways. The natural reservoirs of these viruses are wild aquatic birds, which become infectious very quickly after exposure [9,10]. HPAI viral spillover into new areas and susceptible populations occurs primarily from migrating infected wild birds [3,11,12]. Once introduced, the virus usually spreads rapidly within poultry farms through direct contact between birds, the fecal-oral route, or contaminated media (e.g., equipment, clothing) [13,14]. Environmental contamination plays a crucial role in indirect transmission, as HPAI viruses can survive in water, soil, and organic matter, thereby becoming a persistent source of infection [15]. The transportation and trade of poultry and livestock further expand the spread of the virus [16–19] without adequate measures in place [20].

The H5N1 viruses, descendants of the A/goose/Guangdong/1/1996 (Gs/Gd) lineage, including the widely disseminated 2.3.4.4b, have drawn sustained global attention [21]. First detected in poultry in 1996 with human cases reported in Hong Kong in 1997, H5N1 is now entrenched in parts of Asia and Africa and has spread to Europe and North America [22,23]. Since the early 2020s, a sustained H5N1 panzootic has intensified, characterized by rapid geographic expansion, large-scale wild bird mortality, and unprecedented cross-species transmission in North America [24]. In 2024, Canada reported its first domestically acquired human H5N1 infection, coinciding with widespread outbreaks in wild birds, poultry, and increasing reports in mammals, including livestock [25]. In the United States, H5N1 was first detected in dairy cattle in March 2024, and by the end of that year, it was reported in 689 herds across 15 states, with within-herd clinical illness affecting up to 20% of adult cows [26]. Several human cases, including farm workers in Texas and Michigan, were linked to occupational exposure, and by early 2025, over 70 human cases in the Americas were associated with infected dairy cattle [27,28].

Given the severe disease burden in poultry and cattle, and the ongoing concerns about zoonotic transmission to humans, risk regions worldwide have implemented a range of control measures. These include farm quarantine (movement restrictions and isolation on affected premises), strict biosecurity protocols, epidemiological investigations and contact tracing, culling of infected and exposed flocks or herds, safe disposal of animal products, and vaccination [20,29–31]. While culling infected and exposed populations is drastic, it aims to rapidly remove susceptible hosts and reduce selection pressure for viral evolution [32]. However, these measures can have substantial economic and social impacts, highlighting the need to rigorously evaluate their effectiveness across different epidemiological settings and host populations.

Mathematical modelling has become an indispensable tool in infectious disease epidemiology, providing crucial insights into the understanding and management of complex pathogens such as HPAI [33–35]. These models provide a framework to elucidate complex transmission dynamics, such as bird-to-bird and bird-to-mammal transmission pathways, and to assess the risk of viral adaptation and spillover to humans [36,37]. They can also be used to evaluate potential interventions by simulating their effectiveness before implementation, thereby optimizing resource allocation [38]. Mathematical models also help estimate key epidemiological parameters, such as the basic reproduction number (${ℛ}_{0}$) and transmission rates [39,40]. Given the still-limited but increasing number of human H5N1 cases to date, and the inherent challenges in directly observing rare spillover events or potential human-to-human transmission, mathematical models are particularly valuable. They serve as critical tools for understanding unobservable dynamics, assessing future pandemic risks, and informing preparedness strategies in data-sparse scenarios.

We identified two prior reviews on this topic. The first review encompassed both LPAI and HPAI, integrating mathematical, statistical, machine learning, and hybrid modelling approaches [41]. However, its broad scope reduced comparability of mechanistic assumptions and provided limited guidance on parameterization, model validation, or policy application. The second review synthesizes 46 mechanistic studies, with a search cutoff date of April 2023 [42]. It primarily focuses on the transmission of LPAI and HPAI in wild birds and poultry, emphasizing parameter estimation and the evaluation of culling or vaccination strategies. A few studies explicitly addressed avian–human transmission, but livestock populations were absent, the avian–livestock–human interface was not considered, and movement or trade networks were only minimally represented. Since 2023, North America has witnessed several cross-species transmission events, including spillover from infected poultry to dairy cattle and subsequent human cases [43]. These events have prompted a shift in mechanistic modelling toward multi-host, One Health–oriented frameworks that integrate wildlife, poultry, livestock, and human components, often incorporating cross-species empirical parameterization and validation. This shift requires a focused synthesis of recent mechanistic models of HPAI to characterize these emerging approaches and their remaining limitations. Accordingly, we conduct a scoping review of mechanistic HPAI models published between January 2023 and June 2025.

### Objectives

The objectives of this scoping literature review are to:

- Identify and catalogue mechanistic models used to study HPAI transmission dynamics across various host species (e.g., wild birds, poultry, livestock, and humans), with particular focus on H5 and H7 subtypes.
- Summarize the data sources, modelling approaches, study purposes, and key parameters reported in these studies.
- Highlight gaps and limitations in the current literature, particularly in the One Health context, including cross-species transmission, emerging zoonotic risks, and the pandemic potential of HPAI.

## Methods

### Protocol and registration

**Fig 1:**
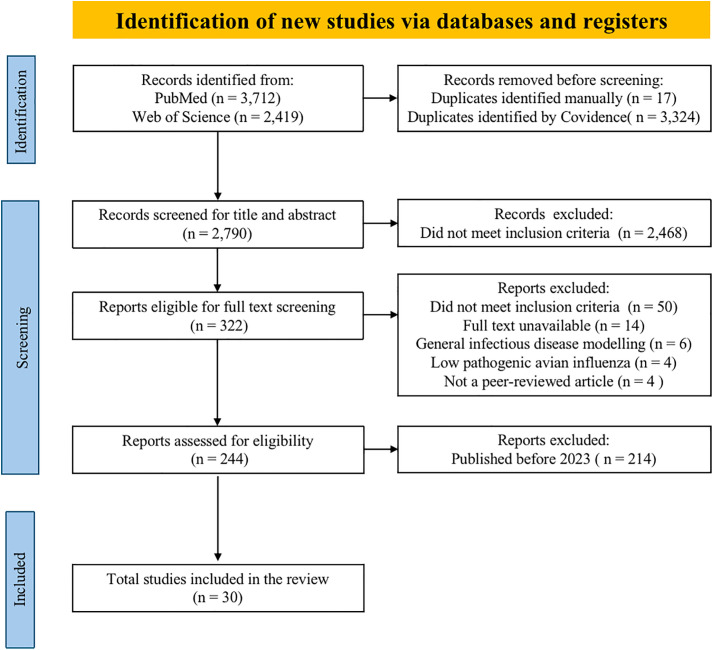
PRISMA flow diagram of identification of new studies.

This scoping review was conducted following the Preferred Reporting Items for Systematic Reviews and Meta-Analyses extension for Scoping Reviews (PRISMA-ScR) guidelines and checklist [44]. All relevant checklist items were included, except for the critical appraisal of individual sources of evidence. The primary goal of this review was to map and categorize existing mechanistic models and associated data and parameters used in studying HPAI transmission dynamics. A completed PRISMA-ScR checklist is provided in Fig 1, and a detailed description of each methodological step is outlined below.

### Information sources

Two databases were searched: PubMed and Web of Science. PubMed was selected for its comprehensive coverage of literature in life sciences and biomedicine, which are central to the focus of this study. To broaden the scope and capture relevant studies beyond the biomedical field, Web of Science was also included as a complementary source.

### Search

The search was conducted on 13 June 2025 using two complementary strategies to maximize retrieval. In Strategy 1, we queried PubMed and Web of Science with Boolean combinations of three keyword sets applied to titles/abstracts/keywords: set 1 = (HPAI OR “avian influenza” OR H5N1); set 2 = (model); set 3 = (mathematical OR math OR equation OR predictive OR surveillance OR forecast). Queries followed the pattern (set 1) AND (set 2) AND (set 3).

Search Strategy 2 broadened the scope by modifying Keyword set 1 to include: HPAI, avian influenza and H5. The same combinations with Keyword sets 2 and 3 were then applied.

Both search strategies were used to query PubMed and Web of Science. The specific implementation of these search strategies for each database is provided in S1 Appendix. The process of determining study eligibility was conducted collaboratively by five authors (MW, EL, TP, JM, BN).

### Eligibility criteria

The following inclusion and exclusion criteria were applied to the study selection process. Studies were included if they met the following criteria: (1) original, peer-reviewed research articles published in English; (2) used mechanistic modelling approaches (compartmental models, agent-based models, networks) to describe the transmission dynamics of the HPAI virus; and (3) focused on transmission between wild birds, poultry farms, livestock farms, or humans. Studies that fell under the following exclusion criteria were rejected: (1) within-host models focusing on immune dynamics or viral evolution of avian influenza; (2) ecological niche models, multi-criteria decision analyses, spatial risk modelling with GIS, machine learning; (3) primarily or solely statistical methods; (4) epidemiological studies; (5) review papers and commentaries; (6) focus on LPAI only; (7) studies published in languages other than English; (8) studies without full text availability. No geographical or date restrictions were applied at the start of this scoping review. However, since a previous scoping review had already synthesized studies up to April 2023 [42], we focused on including studies published between January 2023 and June 2025 to capture the most recent research.

### Selection of sources of evidence

The search results (*n* = 6,131) from each database were uploaded to the scoping review platform Covidence, where duplicates (*n* = 3,341) were automatically removed. In the first round of screening, titles and abstracts of the retrieved papers were reviewed. Four authors (MW, EL, TP, BN) participated in this stage. Three authors (MW, EL, TP) independently assessed each paper, with three possible voting options: ‘yes’, ‘no’, or ‘maybe’. The system required two reviewers per paper. Papers receiving two ‘yes’ votes proceeded to the second round of screening, while those with two ‘no’ votes were removed and marked as ‘irrelevant’. Papers with inconsistent votes, or where one reviewer selected ‘maybe’, were flagged as ‘conflict’ and reviewed by a senior author (BN). The first-round screening specifically assessed whether a paper addressed mechanistic models of avian influenza. Consequently, review articles, experimental studies, machine learning approaches, ecological niche modelling, and multi-criteria decision analyses with GIS were excluded.

The second round of screening was the full-text review. Four authors (MW, EL, TP, BN) participated in this phase. Three authors (MW, EL, TP) independently reviewed each paper, selecting either ‘include’ or ‘exclude’ and providing reasons for exclusion when applicable. The system required two reviewers per paper. Papers with two ‘include’ votes proceeded to the next stage (‘extraction’), while those with two ‘exclude’ votes citing the same reason were removed. If the two reviewers disagreed or provided different exclusion reasons, the paper was marked as ‘conflict’. Conflicted papers were jointly reviewed and discussed by the three authors (MW, EL, TP) to reach a consensus; if consensus could not be achieved, the fourth author (BN) participated in the discussion and made the final decision.

### Data charting process and data items

For each included study, data were extracted into a standardized spreadsheet provided in S1 Table. The charting process captured bibliographic information (electronic link to the publication, year of publication), study characteristics (pathotype, subtype, spatial scale, principal region or country, and epidemic year), and study objectives. Details of the modelling approach were also recorded, including model type, equations, transmission mechanisms, host populations considered (humans, wild birds, poultry, and other animals), control measures, and calibration and validation procedures. We also documented parameter values and sources, data sources and types, and the availability of data and code links. Finally, we extracted key results along with reviewers’ notes on study limitations.

### Synthesis of results

The included studies were organized according to the type of mechanistic model used to study HPAI transmission dynamics. Three main categories were identified. The first category comprised compartmental models, which describe transmission dynamics using differential equations. In the context of HPAI, these models were applied to represent infections across multiple host populations, including wild birds, domestic poultry, livestock, and humans. They were used to explore within- and between-species transmission, assess the impact of interventions such as culling, vaccination, and movement restrictions, and to examine risks of zoonotic spillover. The second category comprised agent-based models, which simulate the behaviours and interactions of individual hosts or farms, often incorporating spatial, ecological, or behavioural heterogeneity. These models capture stochasticity and complex system dynamics emerging from individual-level interactions. The third category comprised network models, which represent hosts, farms, or geographic regions as nodes connected by edges that describe potential transmission pathways, such as movements, trade, or environmental contact. Network models can be deterministic or stochastic, and may integrate features from compartmental or agent-based frameworks. In the context of HPAI, they were used to analyze transmission across poultry transportation systems or to explore stochastic processes and control strategies within networked populations.

## Results

### Selection of sources of evidence

The database search identified 6,131 records. After removing duplicates, 3,341 unique studies entered the screening phase. Following title and abstract screening, 322 studies were retained for full-text review. Of these, 244 met the eligibility criteria. After applying the final inclusion criteria and restricting the date range to studies published after 2023, a total of 30 studies were included in this scoping review. Fig 1 presents a PRISMA flow diagram summarizing the screening and selection process.

### Characteristics of sources of evidence

Table 1 provides an overview of the modelling approaches used in the 30 HPAI studies included in this scoping review, classified by model type: compartmental, agent-based and network models. For compartmental models, we further distinguish between deterministic and stochastic approaches.

**Table 1:** Summary of modelling approaches in 30 HPAI studies included in the scoping review.

Model Type	Method	Number of Models
Compartmental Model	Deterministic	16
	Stochastic	10
Agent-Based Model	Simulation	2
Network Model	Simulation	2
**Total**	–	30

**Fig 2:**
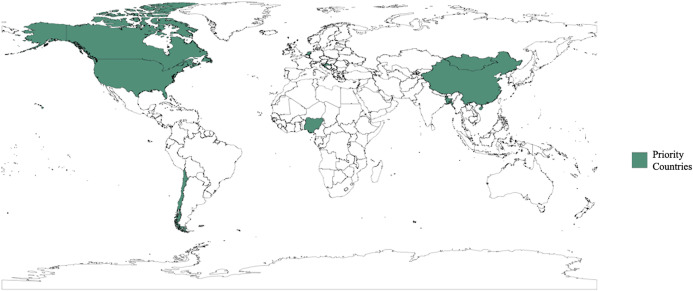
Specific countries are included in the articles (n = 12, 40%). Map generated using the open-source Natural Earth dataset [73] with the R packages ‘rnaturalearth’ and ‘rnaturalearthdata’. Countries reflect the study locations extracted from the included articles.

Based on this classification, Table 2 provides detailed information for each study, including the HPAI subtype(s) considered, the key geographical region(s), the epidemic year(s), the model type, and whether cross-species transmission is included (yes/no). All included studies were published after 2023. Of these studies, twelve (40%) focused on particular countries in the following regions: Europe (*n* = 4, 33.3%), Asia (*n* = 4, 33.3%), the Americas (*n* = 3, 25%), and Africa (*n* = 1, 8.3%). The remaining 18 studies (60%) were location-agnostic (Fig 2). Seventeen studies (56.7%) prioritized specific HPAI subtypes, including H5N1 (*n* = 7, 41.2%), H5N8 (*n* = 1, 5.9%), H5Nx (*n* = 3, 17.6%), H7N9 (*n* = 4, 23.5%) and multiple subtypes (*n* = 2, 11.8%). Fourteen studies (46.7%) incorporated multiple host populations to evaluate cross-species transmission and zoonotic spillover risk.

**Table 2:** Overview of avian influenza models.

Type (clade)	Subtype	Region	Epidemic years	Cross-species^a^	Model type
H5 (2.3.4.4b)	H5N1	United States	2023–2024	No	Compartmental [45]
H5 (2.3.4.4)	H5N8	France	2016–2017	No	Compartmental [46]
	H5Nx	Netherlands; France	2014–2018; 2016–2017; 2020–2022	No	Compartmental [31]
	H5Nx	Mongolia	–	No	Agent-based [47]
H5	H5N1	Chile	–	Yes	Compartmental [33]
	H5N1	China	2022	No	Compartmental [48]
	H5N1	Croatia	2021–2022	Yes	Compartmental [49]
	H5N1	Nigeria	2014–2016	No	Compartmental [50]
	H5Nx	United States; Canada	–	Yes	Compartmental [51]
	H5N1	–	–	No	Compartmental [52]
	H5N1	–	–	No	Compartmental [53]
H7	H7N9	China	2014	No	Compartmental [54]
	H7N9	–	–	Yes	Compartmental [55–57]
Multiple	H5N1; H5N2; H7N7	–	–	No	Agent-based [58]
	H5Nx; H7Nx	–	–	Yes	Compartmental [59]
Unspecified	–	Bangladesh	2016–2018	No	Compartmental [60]
	–	France	–	No	Compartmental [61]
	–	–	–	No	Compartmental [62,63]
	–	–	–	Yes	Compartmental [64–70]
	–	–	–	No	Network [71]
	–	–	–	Yes	Network [72]

^a^No included studies modeled livestock-to-human or avian-to-livestock transmission. Here, ‘cross-species” refers to models that explicitly consider transmission between avian and human populations, as well as between wild birds and poultry.

### Results of individual sources of evidence

The 30 studies included in this review employed three main mechanistic modelling frameworks: compartmental (*n* = 26, approximately 86.7%), agent-based (*n* = 2, approximately 6.7%) and network (*n* = 2, approximately 6.7%) (Table 1 and 2). Compartmental models were the most common, with 16 studies (approximately 61.5%) using deterministic approaches and 10 studies (approximately 38.5%) employing stochastic models. These models were primarily used to describe transmission dynamics among wild birds, poultry, and humans. The optimization and effect of control measures such as culling, vaccination, movement restrictions, and biosecurity interventions were evaluated in 19 studies (63.3%), while 20 studies (66.7%) provided estimates of key epidemiological parameters, including the basic reproduction number (${ℛ}_{0}$).

Agent-based models (*n* = 2, 6.7%) simulated individual-level interactions within and between farms, wildlife populations, and human communities. They captured spatial heterogeneity and heterogeneous contact patterns, allowing for exploration of outbreak scenarios under varying assumptions of host behaviour, farm density, and environmental persistence.

Network models (*n* = 2, 6.7%) represented hosts, farms, markets or geographic regions as interconnected nodes linked by potential transmission pathways such as trade, transportation, or environmental contact. These models were used to study stochastic processes and intervention strategies in networked populations and to simulate disease spread within poultry transportation systems.

Across all modelling frameworks, common findings included the critical importance of rapid detection [45,48,53,61,63], implementation of farm-level biosecurity measures [46,48,53,59], and movement restrictions to limit the spread of HPAI [45,46,50,54,71]. A subset of studies also explored co-circulation of multiple subtypes, co-infections, or cross-species transmission, though these aspects were less frequently addressed [55,56,74].

### Synthesis of results

#### Compartmental models (*n* = 26).

**Fig 3:**
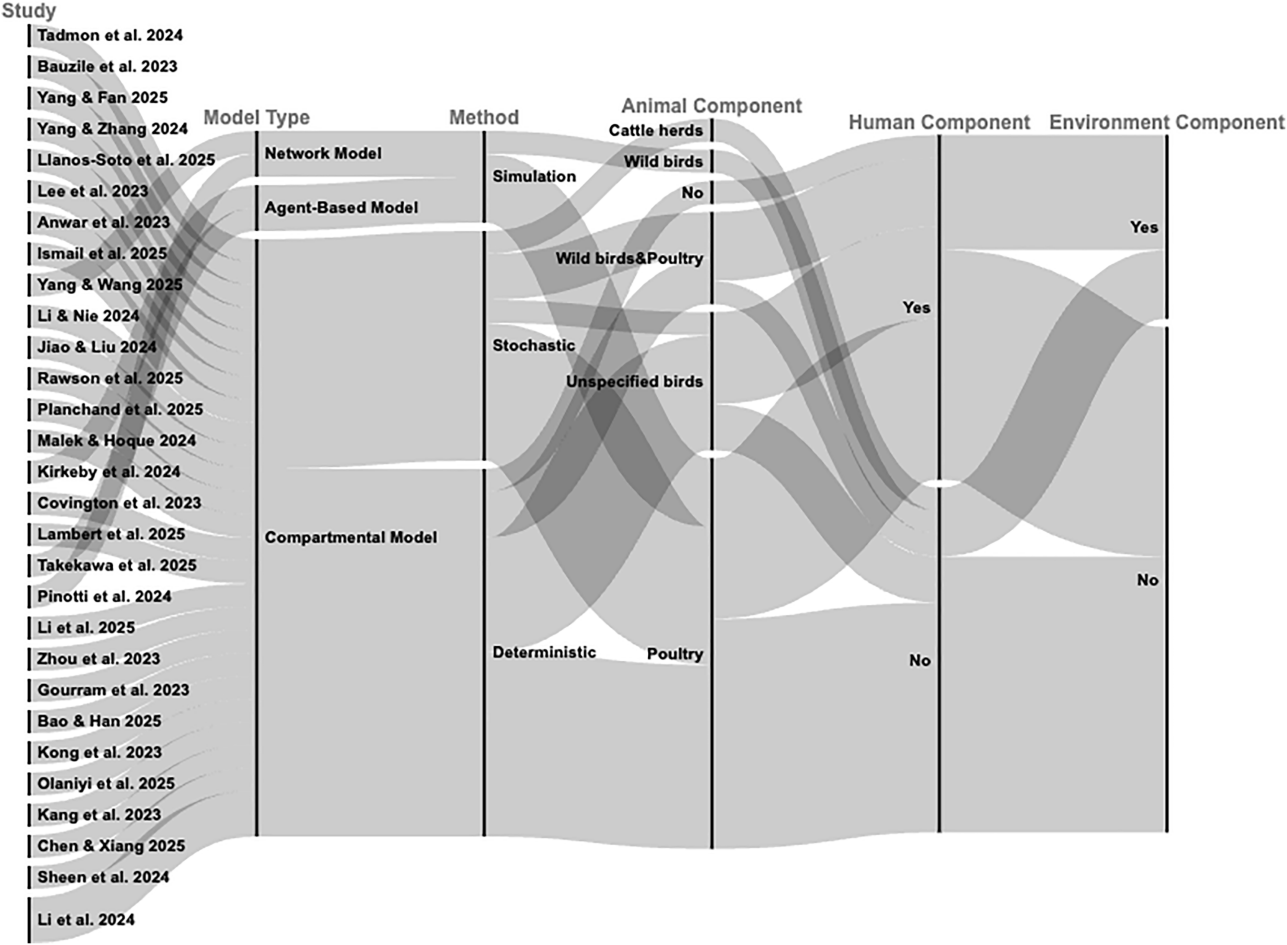
Alluvial diagram depicting the relationships among studies, model types, modelling methods, avian components, human components, and environmental components. The width of each flow is proportional to the number of studies that share the connected attributes.

Among the studies included in this review, 26 studies (86.7%) employed compartmental models to describe the transmission dynamics of HPAI across multiple host populations, including wild birds, poultry, cattle and humans (Table 3). To visualize the relationships among studies, model types, modelling methods, and host or environmental components, we used an alluvial diagram (Fig 3), which highlights how different modelling frameworks capture various aspects of HPAI transmission and the proportion of studies associated with each category.

**Table 3:** Model type, epidemiological host and scale of transmission of the 30 studies.

Model Type	Epidemiological host	Scale of transmission	Area	References
Compartmental (*n* = 26)	Unspecified birds (*n* = 2)	Well-mixed (*n* = 2)	–	[62,72]
	Poultry (*n* = 8)	Well-mixed (*n* = 1)	Poultry house	[31]
		Well-mixed (*n* = 1)	China	[48]
		Well-mixed (*n* = 1)	France	[61]
		Well-mixed (*n* = 1)	Rural markets	[53]
		Within-farm (*n* = 1)	China	[54]
		Between-area (*n* = 1)	Nigeria (northern and southern)	[50]
		Between-farms (*n* = 1)	France	[46]
		Between-farms (*n* = 1)	–	[60]
	Cattle herds (*n* = 1)	Between-herds (*n* = 1)	Continental US	[45]
	Human (*n* = 1)	Well-mixed (*n* = 1)	–	[52]
	Wild birds and poultry (*n* = 1)	Well-mixed (*n* = 1)	Croatia	[49]
	Unspecified birds and human (*n* = 4)	Well-mixed (*n* = 3)	–	[33,57,65,69]
		Well-mixed (*n* = 1)	Chile	[33]
	Wild birds and human (*n* = 1)	Well-mixed (*n* = 1)	–	[66]
	Poultry and human (*n* = 6)	Well-mixed (*n* = 6)	–	[55,56,59,64,68,70]
	Wild birds, poultry and human (*n* = 2)	Well-mixed (*n* = 1)	–	[67]
		Well-mixed (*n* = 1)	North America	[51]
Agent-based (*n* = 2)	Poultry (*n* = 1)	Well-mixed (*n* = 1)	–	[58]
	Wild birds (*n* = 1)	Well-mixed (*n* = 1)	Mongolia	[47]
Network (*n* = 2)	Poultry and human (*n* = 1)	Well-mixed (*n* = 1)	–	[72]
	Poultry (*n* = 1)	Well-mixed (*n* = 1)	Farms and markets	[71]

**Fig 4:**
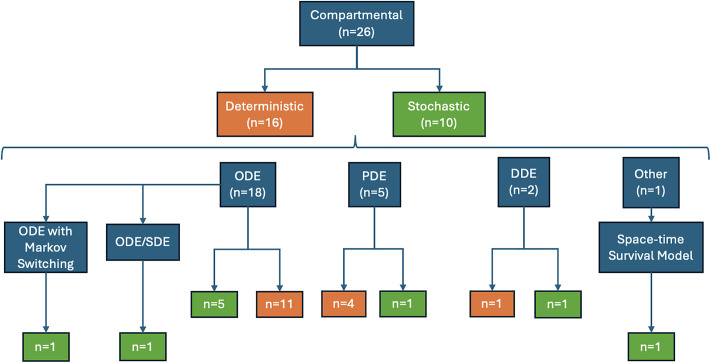
Flowchart of compartmental model types used to model HPAI transmission in the included articles.

**Fig 5:**
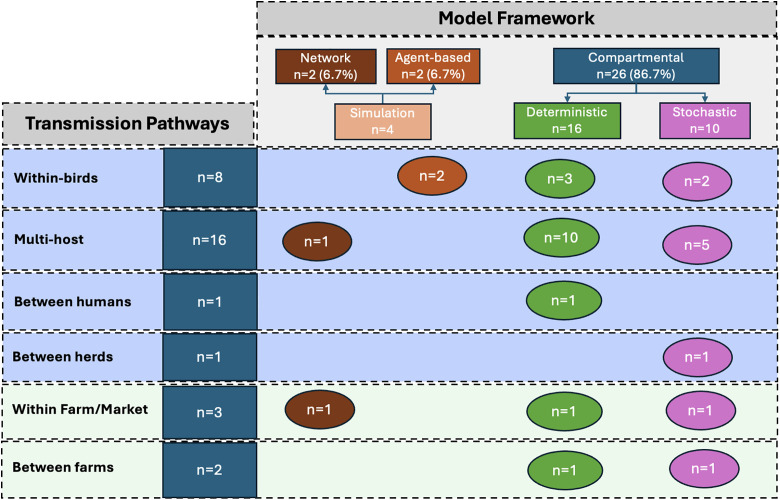
Summary of all HPAI transmission pathways considered according to each modelling framework used in the included studies.

Of these 26 studies, 16 (61.5%) used deterministic formulations, while 10 (38.5%) adopted stochastic frameworks. The methodological distribution, including ordinary differential equations (ODEs), partial differential equations (PDEs), and delay-differential equations (DDEs), is summarized in Fig 4. Six studies (23.1%) incorporated time delays to capture incubation periods, nonlocal transmission, or immunization effects, using stochastic, neural network, or reaction–diffusion frameworks to examine outbreak dynamics, system stability, and the effectiveness of intervention strategies. Regarding host populations, most studies focused on poultry (*n* = 8, 30.8%), poultry–human systems (*n* = 6, 23.1%), wild birds and humans (*n* = 5, 19.2%), or wild birds, poultry, and humans (*n* = 2, 7.7%). Other studies examined wild birds only (*n* = 2, 7.7%), livestock (*n* = 1, 3.8%), or human-only populations (*n* = 1, 3.8%). Environmental compartments representing viral persistence in water, fomites, or other reservoirs were included in eight studies (30.8%). In terms of transmission scale, models spanned within-farm dynamics (*n* = 1, 3.8%), between-farms spread (*n* = 2, 7.7%), between-herds spread (*n* = 1, 3.8%), market-level interactions (*n* = 1, 3.8%), within-species and between-host transmission (*n* = 21, 80.8%), reflecting the diverse pathways through which HPAI can spread across species and production systems (Table 3, Fig 5).

The reviewed compartmental models were generally formulated using standard epidemiological frameworks such as susceptible–infected (SI), susceptible–infected–recovered (SIR), and susceptible–exposed–infected–recovered (SEIR) structures, with extensions tailored to the biology of HPAI. For instance, SI-type models were applied to describe continuous transmission dynamics without recovery, particularly for avian hosts with high mortality [56,63,67]. SIR frameworks, representing infection and subsequent immunity, were commonly used in poultry and mixed-species systems [52,54,65,68]. SEIR-type formulations, which include a latent (exposed) compartment to capture incubation delays, were employed to model temporal progression and environmental persistence of HPAI in both poultry and wild birds [45,46,61,69].

Many studies further introduced additional compartments to reflect interventions or host-specific epidemiological features. Vaccinated or immunized individuals (V/P) were included to capture the effects of vaccination [48,59], while quarantine (Q) and treatment (T) compartments were incorporated to model control strategies [48,60]. Mortality (D) and environmental reservoirs (C/W) were included to represent host death and persistence of the virus in water or on fomites [49,62]. Asymptomatic (A) and clinically ill or hospitalized individuals (H/C) were added to account for subclinical infections and human-specific disease outcomes [33,66]. Subpopulation stratification, for example, by species (wild birds, poultry, humans), resident versus migratory birds, or different herds and market locations, was also common [45,48,53].

**Fig 6:**
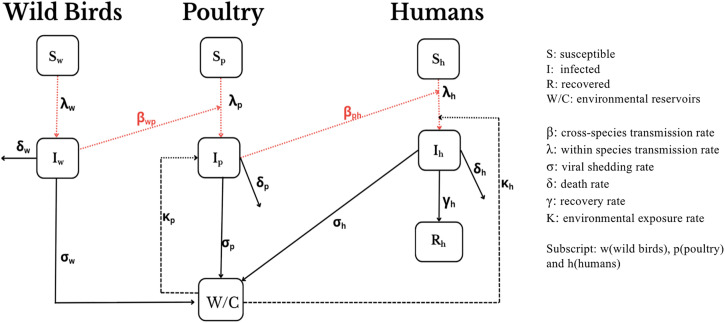
Representative multi-host, compartmental model structure for HPAI transmission.

Fig 6 illustrates representative examples of these model structures: an SI framework for wild birds, an SI-W/C model for poultry (W/C represents environmental viral concentration), and an SIR framework for humans. The diagram shows the flow of infection among three host populations: wild birds (*S*_*w*_, *I*_*w*_), poultry (*S*_*p*_, *I*_*p*_), and humans (*S*_*h*_, *I*_*h*_, *R*_*h*_), as well as the environmental viral concentration (W/C). Subscripts *w*, *p*, and *h* denote wild birds, poultry, and humans, respectively. Transmission occurs within species at a rate λ and across species via explicit cross-species rates ($\beta_{wp}$ from wild birds to poultry, and $\beta_{ph}$ from poultry to humans). The environmental compartment (W/C) introduces a feedback loop, capturing viral shedding from infected hosts ($\sigma_{w},\sigma_{p},\sigma_{h}$) and subsequent environmental exposure, modelled as a transmission rate from the environment to poultry ($\sigma_{p}$) and humans ($\sigma_{h}$). Host-specific epidemiological parameters include the death rate (δ) and, for humans, the recovery rate (γ).

Deterministic models (*n* = 16) primarily focused on threshold analysis and long-term epidemic outcomes. Several studies examined the stability of disease-free and endemic equilibria and derived expressions for the basic reproduction number (*R*_0_), identifying conditions for elimination (*R*_0_ < 1) or persistence (*R*_0_ > 1) [52,54,56,57,59,67]. Some deterministic models (*n* = 13, 81.3%) further incorporated intervention strategies such as culling (*n* = 7, 43.8%), vaccination(*n* = 8, 50%), and quarantine(*n* = 3, 18.8%), showing how their effectiveness depends on host demography and epidemiological context [48,54,57,65,69]. These intervention analyses ranged from theoretical optimal control formulations that identify mathematically optimal intervention trajectories under idealized assumptions, to simulation-based evaluations of fixed or discrete policies intended to reflect operational decision-making. Empirical data, including outbreak reports and mortality records, were also used to calibrate and validate model predictions (Tables 4 and 5). However, according to Table 5, only two studies explicitly validated their model predictions with these data [49,60].

**Table 4:** Summary of empirical data sources used for model calibration and validation in the studies reviewed.

Data source	Region	Year(s)	Reference
Reported cumulative number of bird infections	Nigeria	2014–2016	[50]
Daily mortality data from layer, broiler, and breeder chickens, Pekin ducks, and turkeys	Netherlands	2014–2018; 2020–2021	[31]
Daily mortality data from layer, broiler, and breeder chickens, and mule ducks	France	2016–2017; 2020–2022	[31]
Historical records of H5N1 outbreaks in backyard poultry farms	Croatia	2021–2022	[49]
Observed spatio-temporal distribution of HPAI outbreaks	France	2016–2017	[46]
Farm-level data on production practices and biosecurity measures	China	2022	[48]
The field data of new avian influenza cases	Bangladesh	2016-2018	[60]

**Table 5:** Summary of the 30 included studies.

	Studies	Type of study	Objectives	Modelling approach	Results	Intervention	Optimal control strategy	Validation	Reported limitations
1	Anwar et al. 2023 [68]	Theoretical	To use a backpropagated Bayesian regularization neural network (BBR-NN) to solve a nonlinear delayed avian influenza epidemic model, and evaluate its accuracy and consistency against Adams-solver (ADS) benchmarks across parameter-variation scenarios.	Deterministic delayed compartmental ODE/DDE model of avian–human transmission.	BBR-NN solutions overlap ADS benchmarks across scenarios, with negligible errors. Mean squared error ≈10^−5^ across training, validation, and test sets, with clear convergence. Correlation coefficient *R* ≈ 1 for all datasets.	No	No	No	None reported
2	Chen & Xiang 2025 [52]	Theoretical	Develop and analyse a mathematical model for H5N1 transmission that incorporates a non-linear incidence rate, media reporting, and control measures.	Deterministic compartmental ODE model with nonlinear incidence; optimal control via Pontryagin.	Threshold: DFE stable if ${ℛ}_{0}<1$; endemic if ${ℛ}_{0}>1$. Combination of control measures gives the largest infection reduction; transmission control alone is most economical but insufficient.	Yes	Yes	No	None reported
3	Covington et al. 2023 [62]	Theoretical	Establish uniform persistence for partially diffusive epidemic PDE systems for AIV and Ebola under weaker initial-data assumptions.	Deterministic compartmental reaction–diffusion model with one non-diffusive equation (partially diffusive PDE).	(1) Persistence for ${ℛ}_{0}>1$ if any infection-related compartment starts >0; (2) New argument proving positivity propagation from non-diffusive equations; (3) No improvement to positive steady-state existence.	No	No	No	None reported
4	Gourram et al. 2023 [33]	Applied	Identify effective H5N1 control strategies to reduce severe cases, using optimal control.	Deterministic compartmental ODE model with three time dependent controls.	(1) Awareness and prevention lower infections and treatment follow-up increases treatment among severe cases. (2) Optimally scheduled controls are effective in reducing disease burden (valdted by simulations).	Yes	Yes	No	None reported
5	Ismail et al. 2025 [57]	Applied and Theoretical	Evaluate the impact of avian culling and design optimal control in a delayed avian–human model and analyze stability, and Hopf bifurcation.	Deterministic SI-SEIR compartmental model with logistic growth and time delays.	(1) Hopf bifurcation yields oscillations when delays vary. (2) higher avian carrying capacity *k* and lower bird natural death keep virus persistent. (3) Birds culling are sufficient to reach the disease-free state.	Yes	Yes	No	None reported.
6	Jiao & Liu 2024 [63]	Theoretical	Develop and analyse a delayed avian Filippov model with two culling thresholds to assess how thresholds and delay affect equilibria, sliding dynamics, bifurcations, and control robustness.	Deterministic compartmental 2-D avian SI Fillipov with delay and two thresholds.	(1) Depending on thresholds and delay, solutions converge to a regular equilibrium, a pseudoequilibrium, or a stable periodic orbit; Hopf bifurcation occurs. (2) Time delay strongly affects the sliding mode while increasing delay yields global bifurcations. (3) Two-threshold Filippov control reduces infected birds in many cases, but delay can undermine control.	Yes	Yes	No	None reported.
7	Kang et al. 2023 [64]	Applied and Theoretical	Develop adaptive controls for an avian–human influenza model; prove stability and verify in simulations.	Deterministic compartmental ODE SI-SEIR model.	(1) The system stays stable; poultry numbers and human infection levels converge to their target levels under the designed controllers. (2) Optimal control reduce infection peak. (3) Performance remains effective even when parameters are misspecified	Yes	Yes	No	This study only considers a deterministic model and does not include time delays and stochastic noise.
8	Lee et al. 2023 [50]	Applied	Evaluate and optimize three containment strategies for domestic poultry.	A compartmental SID deterministic ODE model for poultry populations in two regions coupled by a transportation matrix.	(1) A cut (20%) in the first wave rapidly contains transmission and averts the second wave; (2) one-time culling is weaker, while periodic culling can suppress the second wave; (3) Transport reduction between the two regions is least effective.	Yes	Yes	No	Need for finer-granularity transport data.
9	Li 2024 [67]	Theoretical	Prove global stability of the disease-free equilibrium at ${ℛ}_{0}=1$ for a degenerate-diffusion avian-influenza model with seasonality and spatial heterogeneity	Deterministic reaction-diffusion PDE with environmental transmission, spatially heterogenous and partial diffusion.	(1) DFE is globally asymptotically stable at ${ℛ}_{0}=1$; (2) The necessary spectral conditions at this threshold are satisfied, ensuring convergence; (3) All infected populations go to zero; susceptible populations approach spatial steady distributions.	No	No	No	None reported.
10	Li & Nie 2024 [59]	Theoretical	Develop a non-local delayed reaction-diffusion avian-influenza model with vaccination and multiple trasnmission routes and evaluate spatio-temporal effects.	Deterministic compartmental reaction-diffusion PDE model with human and poultry incubation delays on a heterogeneous domain.	(1) If ${ℛ}_{0}<1$ the disease-free equilibrium is globally asymptotically stable; if ${ℛ}_{0}>1$,disease is uniformly persistent; (2) Longer incubation and higher diffusion homogenize spread; (3) Control measures mitigate outbreaks.	No	No	No	Assumptions of fixed spatial boundaries. Seasonality was also not considered in the model.
11	Li et al. 2025 [48]	Applied	Quantitatively assess HPAI spread and mortality under China’s compulsory vaccination schedule in a single henhouse, accounting for maternal antibodies, initial viral load, and transmissibility.	Deterministic compartmental ODE model for HPAI.	(1) With compulsory vaccination, deaths stayed 0.08–0.42.% for moderate spread and even if spread doubled, deaths rose but < 5%; (2) Maternal antibodies were strongly protective: direct bird-to-bird contact drove spread more than the environment; (3) Hitting >70% antibody-poisitive birds and vaccinating on schedule were esential to maintain losses low.	Yes	Yes	No	Key parameters (vaccine protection, awarenes) were unavailable; used proxy immunity data from other H5N1 inactivated chicken vaccines.
12	Malek & Hoque 2024 [60]	Theoretical and Applied	Develop and analyse a mathematical model to examine seasonality and vaccine efficacy shape avian influenza transmission.	Deterministic SEIATR compartmental model with seasonality and nonautonomous analysis.	(1) Higher seasonality and vaccine inefficacy increase outbreak amplitudes and infection waves. (2) Existence of at least one poistive periodic solution when ${ℛ}_{0}>1$. (3) Period-doubling appears as seasonality strengthens.	Yes	Yes	Yes (Quanlitative)	None reported.
13	Olaniyi et al. 2025 [65]	Theoretical and Applied	Examine AI transmission with education-structured susceptibles and isolation-structured infectives.	Deterministic compartmental ODE model.	(1) Effective reproduction number (${ℛ}_{e}$) derived, with global asymptotic stability established for both influenza-free and endemic states; (2) Optimal control strategies considered: awareness, vaccination, treatment and culling; (3) Combinations of any three control measures outperform single interventions and further reduce transmission.	Yes	Yes	No	None reported.
14	Sheen et al. 2024 [53]	Applied	Test whether live-bird markets shift selection toward higher virulence and quantify effects of fast turnover and environmental persistence using an ${ℛ}_{0}$-based evolutionary (ESS) analysis.	Deterministic compartmental ODE model with environmental transmission and migration between farms and markets	(1) Markets select higher virulence than farms; (2) Cleaning lowers selected virulence in markets while with all-in-all cohorts it can instead increase virulence; (3) Moving from markets consistenly reduces the optimal virulence and markets can act as sources of more virulent strains.	No	No	No	Higher market contact rates just scales ${ℛ}_{0}$ and don’t change the selected virulence. Coinfections could alter selection since results may differ with multiple infections.
15	Tadmon et al. 2024 [56]	Theoretical and Applied	Formulate a two-strain avian-human influenza model with environmental transmission and mutation.	Deterministic compartmental ODE model.	(1) Disease-free state if poultry ${ℛ}_{0}<1$ and human ${ℛ}_{0}>1$. (2) The most effective strategy for controlling avian influenza in both populations is vaccination of poultry, while treatment and education are effective measures for the human population. (3) The most cost-effective measure was found to be quarantine of infected human cases when viral mutation is considered.	Yes	Yes	No	Did not model implementation delays in controls. Interventions costs were assumed equals.
16	Yang & Fan 2025 [51]	Theoretical and Applied	Develop a reaction–advection–diffusion AI model with migratory wild birds.	Deterministic spatially structured PDE model (reaction–advection–diffusion).	(1) ${ℛ}_{0}$ is most influenced by wild-bird parameters; (2) spatial poultry infection patterns match observations; (3) Spillover risk varies with bird behavior and impacts poultry more than humans.	No	No	No	Assumes constant wild-bird population and constant convection rate.
17	Bao & Han 2025 [70]	Theoretical	Derive extinction conditions for a stochastic, time-delay AIV model and validate them with a Legendre Spectral collocation method (LSCM).	Stochastic time-delay compartmental model with nonlinear incidence, incorporating random fluctuations (white noise) to capture environmental and transmission uncertainties.	(1)The LSCM approach provides more accurate numerical simulations with smaller errors compared to the Euler method; (2)Sufficient conditions for pathogen extinction in both human and avian populations were also determined for the stochastic model.	No	No	No	None reported
18	Bauzile et al. 2023 [46]	Applied	Quantify how reducing palmiped farm density in the densest municipalities changes ${ℛ}_{0}$ and ${ℛ}_{e}$.	Stochastic spatial farm-based compartmental model.	Reducing farm density of palmipeds led to a reduction in ${ℛ}_{0}$ and epidemic magnitude; however, this measure would not have been effective at mitigating and preventing viral transmission, even with additional the control measures.	Yes	Yes	No	Assumed constant intervention timelness across scenarioswhich may underestimates benefits; External infection ressure constatnt which likely overstimates cases.
19	Kong et al. 2023 [55]	Theoretical	Develop and analyze a stochastic avian influenza model with two pathogenic virus strains to study transmission from poultry to humans in a random environment.	Stochastic compartmental model SDE extension with white noise representing environmental randomness in avian-to-human influenza transmission.	(1) The system settles into a stable endemic pattern when threshold is exceeded with small noise; (2) Higher environmental noise levels can push the systm toward extinction despite deterministic modele persistence; (3) Simulations confirm the theory illustrating the role of stochasticity	No	No	No	Parameters assumed independent; only one type of stochastic process (white noise) used; model not fitted to real outbreak data.
20	Lambert et al. 2025 [31]	Applied and theoretical	Estimate per-flock infection onset (*t*_0_) to guide tracing and evaluate sequential Approximate bayesian Computation (ABC) versus single-pass ABC-rejection, and also provide a deployable Shiny interface.	Stochastic SEIRD compartmental model with probabilistic infection dynamics and Bayesian parameter estimation.	(1) Infection onset (*t*_0_) typically ocurred ∼ 3–20 days and varies across poultry population. (2) Good fit in 61/63 flocks. (3) ABC-rejection produce *t*_0_ close to ABC in minutes, and the fied 21 day tracing can over- or under-shoot	Yes	Yes	No	Limited identifiability with model best fitting unvaccinated, mortality-driven detections; Assumed homogenous mixing and the results are prior-sensitive; defaults to 21 days tracinf if estimates unstable.
21	Li et al. 2024 [54]	Theoretical	Evaluate control strategy (quarantine, vaccination and elimination) under environmental variability.	Stochastic SIQR compartmental model with general incidence and Markov switching.	(1) Derives ${ℛ}_{0}$ threshold and ${ℛ}_{0}>1$ an ergodic stationary distribution exists; (2) Environmental switching drives multi-waves outbreaks even under control; (3) Quarantine and hybrid vaccination–elimination strategies effectively reduce transmission.	Yes	Yes	No	None reported
22	Llanos-Soto et al. 2025 [49]	Applied	Assess reservoir waterfowl stopover duration affects the probability of HPAI infection in backyard poultry farms.	Stochastic compartmental ODE model for HPAI transmission.	(1)Longer mallard stopovers increased backyard-farm infection risk; (2) lower virulence strains with weker immunity increase infection risk; (3) Swans are useful sentinel but low virulence delays die-offs.	No	No	Yes (Quantitative)	Lack of data unable to incorporate co-circulation, heterosubtypic immunity and age structure was not modeled; the results are tied to the modeled species and the sites.
23	Planchand et al. 2025 [61]	Applied	Evaluate sensitivity and alert delay of surveillance options in vaccinated flocks.	Stochastic compartmental SEIRD model for within-flock HPAI virus transmission.	Enhanced passive surveillance (samples tested from dead ducks, weekly) was the most sensitive (∼ 81% − 88%) and timely approach with regards to viral detection.	Yes	No	No	Other key sampling approaches (e.g., environmental samples) or diagnostic methods beyond RT-PCR were not considered.
24	Rawson et al. 2025 [45]	Applied	Estimate under-reporting H5N1 transmission in livestock by state-level, forecast export-test positivity over time and test border-testing counterfactuals.	Stochastic metatpopulation compartmental SEIR model	(1) The model estimated underreported H5N1 cases; (2) Outbreaks are likely to be reporte in states with large dairy hers (West Coast); (3) Current rules only prevented about 175 reported outbreaks and not enough to change the overall trajectory; (4) The outbreak dynamics can change without stronger farm-level biosecurity.	Yes	Yes	No	Strong assumptions on ascertainment due to sparse data including probabilistic methods to estimate 2024 cattle movements.
25	Yang & Zhang 2024 [66]	Theoretical	Characterize stability of an avian-influenza system with space and delay and design a fixed-time controller that guarantees stabilization within a preset time.	Stochastic compartmental ODE model with spatial diffusion and nonlocal delay.	(1) Stability proven decay to zero in mean-square and almost-sure exponential senses; (2) A fixed-time controller with settling time set by gains and independent of initial state; (3) Numerical simulations display rapid convergence consistent with theory.	Yes	Yes	Yes	Random structures such as age structures, impulsive perturbations, Markov switching, and more are not modeled.
26	Zhou et al. 2023 [69]	Theoretical and Applied	Derive a threshold *R*_*o*_ and a stationary distribution and assess how noise and behaviour affect persistence and extinction in AIV transmission.	Stochastic compartmental ODE model with nonlinear incidence.	(1) Threshold ${ℛ}_{0}$ proven (persistence with unique stationary distribution if >1); (2) “Psychological response” reduces human infections but not vian risk; (3) Noise enters ${ℛ}_{0}$ and critically shapes dynamics which is confirmed by numerical simulations.	No	No	No	None reported
27	Kirkeby et al. 2024 [58]	Theoretical	Quantify species- and subtype-specific differences in outbreak speed and severity to guide surveillance design.	Stochastic individual-based SEIR (agent-based) model	(1) Outbreak duration and severity vary by species and subtype; (2) In poultry, HPAI is shorter than LPAI; (3) In chickens H5N1/H7N1/H7N3 are most severe while H5N2/H7N7 are less severe; (4) Surveillance must be more sensitive and timely for some subtypes to allow control actions in time.	Yes	Yes	No	Missing latency data for some subtypes leading to the use of a default 1-day latency; Species coverage limited to chickens and turkeys only; parameters are literature-based and distributions are assumed normal.
28	Takekawa et al. 2025 [47]	Applied	Assess how waterfowl (Swan Geese) molting-season movements and the wetland mosaic shape HPAI spread and persistence	Stochastic individual-based SEIR (agent-based) model.	(1) Persistence tracks molting-season inter-wetland movement and transmission rate; (2) drought clusters birds and brings earlier, larger, deadlier outbreaks without sustaining spread; (3) Higher transmissibility (β) yields short, severe outbreaks; (4) Maintaining multiple undisturbed molting wetlands can dilute risk.	No	No	No	Data may not accurately reflect the full range of Swan Geese movements; Study does not account for the potential transmission contribution of other waterbird species within the region.
29	Pinotti et al. 2024 [71]	Applied	Stochastic agent-based SEIR network model (EPINEST)	Assess how heterogenous poultry production-distribution networks shape pathogen spread and mixing, and to run scenario analyses across different structures.	(1) Dense, less-hierarchical vendor movements and higher inter-farm transmission increase strain richness and overlap across live-bird markets; (2) Purely market-driven amplification is limited by the latent period and short marketing times; (3) Transport and farm spread are key for mixing and persistence of AIV spread.	No	No	No	Computational complexity forced many assumptions and simplifications such as fixed behavioural parameters, assuming homogeneous poultry mixing, and excluding environmental transmission risk.
30	Yang & Wang 2025 [72]	Theoretical	Compare optimal versus near-optimal control policies (poultry culling and human treatment) for reducing infections and costs in an avian-human system with delays and spatial spread.	Stochastic Network-based compartmental model with time delays and reaction–diffusion between poultry and human subnetworks.	(1) Despite optimal and near-optimal controls pushing down infection risk, near-optimal is slightly effective but cheaper; (2) Optimal policy suggests short, high-intensity culling (∼7 days) plus timely human treatment (∼14 days); (3) Delay analyses show incubation lags shape trajectories and can sustain human cases longer.	Yes	Yes	No	Noise perturbation is only considered in mortality rate; environmental shocks with Lévy noise not modeled.

Stochastic compartmental models (*n* = 10) incorporated random processes to capture temporal heterogeneity and variability in HPAI transmission dynamics. These processes included stochastic infection events and random selection of farms for removal [46], random perturbations in natural death rates of birds and humans (white noise) [66], stochastic fluctuations in transmission and disease progression [69,70], stochastic regime-switching of transmission rates via a Markov process [54], and probabilistic infection dynamics and parameter uncertainty modeled through Monte Carlo simulations or Bayesian inference [31,45,49,55,61]. By explicitly modelling these stochastic components, the models capture the inherent randomness in outbreak progression, movement, and detection, which can lead to substantial variation in epidemic size, extinction probability, and outbreak timing, particularly in small populations or during early stages of an epidemic. Among these stochastic models, five studies (50%) evaluated interventions such as culling (*n* = 2, 20%), vaccination (*n* = 2, 20%), and environmental management (*n* = 2, 20%), providing quantitative insights into the effectiveness and uncertainty of control strategies [61,66].

In addition to structural features, quantitative assumptions regarding transmission rates and epidemiological parameters were systematically reported across studies. Table 6 summarizes the transmission rates used in mechanistic HPAI models across different pathways (avian-to-avian, avian-to-human, human-to-human, and environmental transmission). Table 7 further synthesizes selected parameter values for different hosts, including latent and infectious periods, recovery rates, mortality rates, and intervention-related parameters such as culling rates. Together, these tables highlight both the analytical strengths of deterministic models and the probabilistic insights from stochastic models, illustrating their complementary contributions to understanding HPAI dynamics.

**Table 6:** Transmission rates used in mechanistic HPAI models.

Transmission pathway	Transmission rate
**Avian-to-avian**	
Wild birds-to-wild birds	0.25^a^ [33], 2 × 10^−6^ day^−1^ [65]
Wild birds-to-poultry	5.21 × 10^−7^ day^−1^ [49]
Poultry-to-poultry	8.1 × 10^−6^ ∼ 5.1 × 10^−4^ day^−1^ [64,66,71,72]
**Avian-to-human**	
Poultry-to-human	1.54 × 10^−7^ ∼ 4.76 × 10^−3^ day^−1^ [64,66,72,75]
Wild birds-to-human	0.2 ∼ 0.23 [33,57]
**Human-to-human**	1.5 × 10^−8^ ∼ 6 × 10^−7^ day^−1^ [65,72], 0.003 (ind week)^−1^ [56], 0.2 ∼ 0.25^a^ [33,57]
**Environment**	
Environment-to-wild birds	1 × 10^−10^ day^−1^ [49]
Environment-to-poultry	2 × 10^−4^ ∼ 3 × 10^−4^ day^−1^ [48]

^a^ No units reported.

**Table 7:** Selected parameters used in compartmental models of HPAI.

Parameter	Value (source)
Duration of latent period	0.25 ∼ 5 days [31,48,53,71]
Duration of infectious period	2 ∼ 5.5 days [48,53,71]
Death rate of birds due to infection	0.7 ∼ 1^a^ [60]; 0.0025 ∼ 0.015 day^−1^ [55,64,66,68]
Death rate of humans due to infection	0.001 ∼ 0.3445 day^−1^ [51,55,57,64,66,68]
Recovery rate of asymptomatic poultry	0.6 [60]
Recovery rate of symptomatic poultry	0 ∼ 0.1^a^ [60]
Recovery rate of humans	0.01 ∼ 0.61^a^ [51,55,57,64–66,69]
Average number of viruses released into the environment by infectious wild birds	5 week^−1^ [51]
Average number of viruses released into the environment by infectious poultry	15 week^−1^ [51]
Culling rate of infected birds	0.2^a^ [57]
Culling rate of infected poultry farms	0.29 farm day^−1^ [49]

^a^No units reported.

#### Agent-based Model (*n* = 2).

Agent-based models (ABMs) simulate the behaviours and interactions of individual hosts or entities to capture emergent epidemic dynamics in complex systems. Unlike deterministic compartmental models, ABMs allow for explicit representation of host heterogeneity, stochasticity, movement patterns, and contact structures, making them particularly suited for exploring fine-scale processes in HPAI transmission across poultry populations, wildlife, and farm networks.

Two studies applied ABMs to investigate HPAI transmission dynamics. Kirkeby et al. [58] developed an individual-based SEIR model to simulate outbreaks in large poultry flocks under different virus subtypes (H5 and H7) and poultry species. Using reported parameter estimates and literature-based assumptions, the model highlighted considerable differences in outbreak size and duration between species and subtypes, with implications for surveillance design and outbreak preparedness. A key limitation identified was the lack of detailed latency period estimates across genotypes, particularly for dominant strains such as H5N8. Takekawa et al. [47] constructed an ABM informed by telemetry data on Swan Geese in northeastern Mongolia. Their model linked host movement, environmental landscapes, and pathogen parameters to assess the likelihood of HPAI persistence during the moulting and pre-migration periods. Results emphasized that conserving undisturbed habitats for wild waterfowl could reduce transmission risk, underscoring the value of coupling ecological data with epidemiological simulations.

These two ABMs extend HPAI modelling beyond compartmental approaches by incorporating host-level variability, spatial movement, and network connectivity. They illustrate the utility of ABMs for evaluating outbreak dynamics in complex production and ecological systems, while also highlighting critical data gaps, particularly in subtype-specific epidemiological parameters. However, ABMs require detailed data on host populations, movement patterns, and environmental landscapes, as well as fine-scale parameter estimates such as latency and transmission rates. They are computationally intensive, often necessitating multiple simulation runs to capture stochastic variability and test different scenarios, which can demand high-performance computing resources, especially for large populations or extended temporal horizons.

#### Network Model (*n* = 2).

Network models capture the structure and connectivity of contacts among hosts, farms, or markets, offering a framework to study how disease transmission is influenced by network topology, mobility, and control interventions. These models are particularly useful for representing heterogeneous and spatially structured systems, where the spread of HPAI depends on complex interactions across trade or contact networks.

Two studies employed network-based approaches to explore avian influenza transmission dynamics. Yang and Wang [72] developed a stochastic reaction–diffusion avian influenza model on complex networks to investigate human–poultry transmission and near-optimal control strategies. The model incorporated multi-time delays and stochasticity to describe the co-circulation of avian and mutant human strains across networked nodes representing poultry populations and human communities. Using Ekeland’s variational principle and the Pontryagin random maximum principle, the authors derived necessary and sufficient conditions for near-optimal interventions, including culling of infected birds and treatment of human cases. Simulation results indicated that high-intensity culling and early treatment could suppress infection peaks, although the analysis was entirely theoretical and not calibrated with real outbreak data.

Pinotti et al. [71] introduced EPINEST (Epidemic Network Simulation in Poultry Transportation Systems), a modelling framework designed to simulate pathogen spread within poultry production and trade networks. The model represented interactions among farms, middlemen, vendors, and live bird markets as nodes in a transportation network, enabling exploration of how trade connectivity affects disease propagation. Simulations demonstrated that network structure and movement patterns strongly influenced outbreak size and duration. While parameter values were partly assumed and model validation was limited, EPINEST highlights the potential of network-based approaches to evaluate targeted control measures, such as trade restrictions or improved biosecurity at key nodes.

These studies demonstrate the value of network models for representing heterogeneous contact structures and identifying critical nodes or links that influence the spread of HPAI across poultry production and human–poultry interfaces. Network models rely on detailed data on contact patterns, trade movements, and farm or market connectivity. Incorporating stochasticity, delays, and intervention strategies increases computational complexity, often requiring repeated simulations to evaluate outbreak scenarios and the effectiveness of control measures. Consequently, these models demand substantial computational resources and careful data curation to ensure realistic network representations.

## Discussion

This scoping review synthesized modelling approaches that have been used to investigate the transmission dynamics of HPAI across wild birds, poultry, livestock, and humans (Table 2 and 5, Figs 3 and 5). Three main methodological traditions were identified: compartmental models (deterministic and stochastic), agent-based models, and network models. Compared with earlier reviews, such as Lambert et al. [42], which included studies up to April 2023 and primarily focused on wild birds and poultry with limited consideration of avian–human transmission, the studies published from January 2023 to June 2025 show notable advances. These recent studies increasingly incorporate multi-host, “One Health”-oriented frameworks that explicitly address interspecies transmission, including the human-animal interface, and, in some cases, livestock populations. They also demonstrate enhanced empirical parameterization and validation. Given the increasing circulation of HPAI and heightened urgency for detection, prediction, mitigation, and prevention of further cross-species transmission and potential human-to-human spread, understanding these recent modelling developments is critical for informing effective outbreak preparedness and risk assessment.

Compartmental models remain the dominant approach in HPAI transmission modelling, largely due to their analytical tractability and capacity to yield threshold quantities such as the basic reproduction number (*R*_0_) [46,50,51,53,60,62,67]. Deterministic formulations were primarily used to examine long-term epidemic behaviour, equilibrium stability, and intervention effectiveness, while stochastic extensions captured variability in outbreak size, extinction probability, and timing through random infection events, demographic perturbations, and probabilistic transmission processes [46,49,61,66,72]. The flexibility of compartmental frameworks was further demonstrated through extensions incorporating latent, asymptomatic, vaccinated, and environmentally mediated states, enabling representation of complex transmission pathways linking poultry, wild birds, and humans [33,59,60,69]. Together, deterministic and stochastic formulations provide complementary insights, balancing analytical clarity with representation of uncertainty under real-world conditions.

Across these studies, model-based analyses consistently indicated that single interventions were insufficient to control HPAI transmission. Reductions in farm density alone lowered reproduction numbers but did not prevent persistence in palmiped systems [46], whereas combined strategies integrating human-targeted measures, treatment, and vaccination in poultry were most effective in reducing infections across host populations [56,65]. Environmental processes, including viral decay and spatial heterogeneity, emerged as key drivers of persistence and spread, particularly when interventions were delayed [59]. Several models further showed that outbreak severity varied substantially by virus subtype and poultry species, highlighting the importance of pathogen-specific parameterization [58].

Agent-based and network models, though less frequently applied, provided important complementary perspectives by explicitly representing heterogeneity, spatial structure, and contact networks. Agent-based models captured species-specific transmission dynamics and ecological drivers such as migration and habitat use, underscoring the value of integrating behavioural and ecological data [47,58]. Network-based approaches emphasized the role of trade connectivity, mobility, and timing of interventions in shaping outbreak trajectories [71,72]. However, both approaches were constrained by data scarcity, computational demands, and simplifying assumptions, limiting their use for large-scale or empirically calibrated policy evaluation.

While many studies evaluated intervention strategies, an important distinction exists between theoretical optimal control frameworks and simulations of operationally realistic policies. Optimal control studies provide valuable conceptual insights into the timing and intensity of interventions under idealized assumptions. Still, they often assume perfect compliance, continuous control, and unconstrained resources, which limits their direct applicability. In contrast, simulation-based studies that assess discrete, implementable measures, such as fixed vaccination strategies, culling radii, movement restrictions, or biosecurity interventions, offer more immediately actionable guidance for decision-makers. Failure to clearly distinguish between these approaches may lead to overinterpretation of theoretical optima as practical policy recommendations.

Advanced computational approaches, including neural networks and LSCM methods, demonstrated high numerical accuracy and reliability in simulating delayed or stochastic HPAI dynamics [68,70], providing promising alternatives for modelling complex epidemic systems. Similarly, the Laplace residual power series (LRPS) method has demonstrated high numerical accuracy and reliability in simulating complex epidemic [76,77]. While developed for general fractional-order systems or other health conditions, these analytical techniques provide promising alternatives for modelling the non-linear coupled initial value problems characteristic of HPAI, offering rapid convergent series approximations with reduced computational resources. Collectively, these studies indicate that HPAI transmission is shaped by a complex interplay of host species, virus subtypes, environmental conditions, spatial heterogeneity, and intervention strategies, with effective mitigation requiring multi-pronged approaches that integrate vaccination, culling, quarantine, and public health measures tailored to specific host-pathogen contexts.

Existing modelling efforts have addressed multiple scales of HPAI transmission, from within-flock and between-farm spread to interactions among wild birds, poultry, and humans. Deterministic compartmental models provide analytical clarity, stochastic and network-based models capture uncertainty and structural heterogeneity, and agent-based models represent individual-level behaviours and spatial complexity. The combined insights from these approaches highlight significant progress in understanding HPAI dynamics while revealing ongoing challenges related to data integration, model validation, and the translation of theoretical findings into actionable control strategies.

Despite these advances, several limitations were evident across the included HPAI modelling studies, which should be considered when interpreting their findings (Table 5). Data limitations were pervasive in many studies. A number of models relied heavily on assumed or literature-based parameters due to the scarcity of empirical data, leading to substantial uncertainty in the parameter values used across studies [31,45,46,48,49]. Key epidemiological parameters, such as latency periods, genotype-specific transmission rates, and vaccine-induced immunity for different poultry species or viral subtypes, were often unavailable or incomplete [45,47–49,58]. In several cases, default or symbolic values were used to fill these gaps [58,67], which may not accurately represent the complex dynamics of natural infections. Furthermore, the lack of standardized reporting for parameter values, including inconsistent or missing units for transmission rates and disease-induced death rates, makes direct comparison between models difficult and limits the reproducibility of reported findings. Data for certain poultry populations, such as geese or backyard flocks, were particularly sparse [31,49], limiting the ability of models to capture heterogeneity in susceptibility or contact patterns. Surveillance and reporting biases further complicated model calibration; for example, underreporting due to differences in sampling intensity, delayed case detection, or incomplete movement records restricted the capacity of models to reflect true outbreak dynamics [45,49]. Such parameter uncertainty raises questions about the reliability and policy relevance of model outputs, underscoring the need for robust empirical parameterization, uncertainty quantification, and ensemble modelling approaches to support evidence-based decision-making for HPAI control.

Beyond parameter uncertainty, formal model validation against empirical data was notably rare. Among the 30 included studies, only two reported any form of validation, either through qualitative comparison with field surveillance data or quantitative validation using historical outbreak records. The absence of validation in the vast majority of studies substantially limits confidence in model-based inferences, particularly when models are used to evaluate cross-species transmission dynamics or intervention effectiveness. Without validation, it remains unclear whether simulated epidemic trajectories adequately capture real-world transmission processes, host interactions, or reporting mechanisms. This widespread lack of empirical validation represents a major limitation of the current HPAI modelling literature and highlights an urgent need for closer integration between mechanistic models and available surveillance and outbreak data.

Structural and methodological simplifications were common across HPAI modelling studies, limiting model realism and practical applicability. Many models assumed homogeneous mixing within host populations, linear environmental exposure, fixed spatial boundaries, or lifelong immunity without waning [51,52]. When vaccination was included, immune protection was typically represented as binary and permanent, with little consideration of waning immunity, heterogeneity in immune responses across individuals or species, or the presence of passive immunity in young animals. Such simplifications overlook experimentally observed variation in vaccine-induced humoral immunity and maternal antibody transfer, which can strongly influence susceptibility, transmission dynamics, and the timing of outbreaks [78].

Deterministic compartmental models can be extended to include multiple host populations, intervention processes, and cross-species interfaces. However, these models typically neglect stochasticity and spatial heterogeneity [56,59], both of which are crucial for capturing variability and localized clustering in real outbreaks. While some studies implemented mechanistic representations of interspecies transmission, explicitly modelling biological interactions between host species, many relied on symbolic or exploratory couplings based on assumed parameters. This distinction is important, as models with weakly parameterized host interactions may provide limited insight into actual cross-species transmission dynamics. To better account for heterogeneity in transmission risks arising from farm organization, animal movements, and host–species interactions, these extended frameworks are often formulated as stochastic spatial or network-based models that incorporate movement networks and spatial diffusion across farms and populations [72]. While agent-based and network models offered greater flexibility in representing individual-level interactions and complex contact structures, their applications were constrained by computational demands, theoretical simplifications, or limited behavioural and empirical data for parameterization and validation [58,71]. Stochastic or delay models typically focus on theoretical aspects, such as incubation periods or delay dynamics, without empirical calibration, reducing their relevance for policy evaluation [31,46].

Furthermore, many studies did not explicitly incorporate key epidemiological processes such as co-infections, spillover from wild birds, or interspecies transmission, which could lead to over- or underestimation of outbreak risk [45,56]. In addition to mechanistic approaches, several studies used statistical or risk-based models to evaluate the likelihood of avian influenza transmission and assess relative risk at the human–animal–environment interface [75,79,80]. These frameworks typically combine spatial density, environmental exposure, and host interaction data to identify high-risk areas or transmission pathways. While such approaches provide valuable insights for targeted surveillance and early warning, they often rely on simplified assumptions or limited spatial and behavioural data, which may constrain their predictive accuracy and generalizability across regions or host systems.

These limitations reveal the challenges of accurately modelling HPAI dynamics. They highlight the critical need for improved empirical data collection across diverse host species and viral genotypes, enhanced surveillance systems that reduce reporting biases, and model designs that incorporate stochasticity, spatial heterogeneity, and realistic interventions. Addressing these gaps will improve the reliability of model predictions, support evidence-based outbreak preparedness, and inform effective policy and control strategies. Notably, despite reported outbreaks in cattle and other livestock, these settings remain largely underrepresented in mechanistic models. Among the 30 included studies, only one specifically modelled cattle, and while several considered humans and poultry farms, none explicitly examined avian–livestock or livestock–human transmission pathways. In particular, the lack of empirically informed immune processes is most pronounced in livestock settings, where age structure, vaccination practices, and passive maternal immunity may substantially shape susceptibility and transmission but are rarely represented in mechanistic models. This highlights a critical gap in modelling livestock populations and cross-species transmission among avian, livestock, and human populations, an area identified as a priority for understanding zoonotic risk. Experimental and observational datasets, such as antibody kinetics, maternal antibody transfer in milk, and seroconversion timelines, offer valuable opportunities to improve mechanistic models. Incorporating these data can help parameterize transition rates between compartments, support age-structured or immunity-stratified frameworks, and reduce reliance on assumed or literature-based [78]. Leveraging such empirical evidence, particularly in livestock populations, can enhance model fidelity, improve biological realism, and strengthen the relevance of models for informing outbreak preparedness and intervention strategies.

In addition to the limitations identified within the included studies, several modelling approaches were excluded due to the defined scope of this scoping review. Within-host models were not considered, as the focus was on population-level transmission; however, they can provide valuable insights into viral replication, immune responses, and evolutionary processes relevant to cross-species spillover. Hybrid approaches linking within-host and between-host dynamics could further enhance understanding of viral adaptation and zoonotic emergence, providing insights into viral evolution and disease progression. GIS-based multi-criteria decision analysis models were also excluded. Although these approaches do not explicitly model transmission dynamics, they offer complementary insights by highlighting spatial heterogeneity and identifying high-risk regions, supporting targeted surveillance and intervention planning. The exclusion of these frameworks reflects the review scope rather than their relevance, and future research could benefit from integrating them with mechanistic transmission models.

## Conclusions

In response to the increasing focus and risk of HPAI virus, our scoping review provides a necessary updated summary of the literature published on HPAI modelling since 2023, extending the results of Lambert et al. [42]. Our study includes compartmental (deterministic and stochastic approaches), agent-based and network modelling approaches, with a focus on outbreak dynamics and intervention assessment. The review revealed that most studies relied on deterministic compartmental models with limited stochasticity or spatial heterogeneity, and often used assumed or literature-based parameters due to gaps in empirical data. Agent-based and delay models offered greater flexibility for incorporating heterogeneity and behavioural dynamics, but were often constrained by computational demands and the limited availability of data for calibration and validation. Network models, though less common, provided valuable insights into how structural connectivity, such as poultry trade routes, market linkages, and contact networks, shapes outbreak propagation and informs targeted control strategies.

Nevertheless, important gaps remain. Key epidemiological processes, including co-infections, heterogeneity across host populations, and interactions between humans and livestock, are frequently underrepresented. Intervention strategies are not consistently incorporated, and many models rely on simplified assumptions that may limit predictive accuracy. In particular, immunity is frequently oversimplified, with most models assuming lifelong or homogeneous protection following vaccination or infection. Future modelling efforts should incorporate empirically informed immune dynamics, including waning immunity, heterogeneous vaccine responses, and passive maternal antibody transfer, drawing on experimental studies of humoral immunity in livestock to improve biological realism and policy relevance [78]. Crucially, formal model validation against empirical outbreak data remains rare. This pervasive lack of validation substantially limits confidence in model predictions and represents a major constraint on the credibility and policy relevance of the current HPAI modelling literature. These limitations highlight the need for improved empirical data collection, model validation, and more realistic modelling frameworks to better inform outbreak preparedness and policy decisions. Based on these findings, future efforts should prioritize the collection of high-quality empirical data to support robust parameterization and model validation, including immunological data on antibody kinetics and maternal antibody transfer, to inform immunity-stratified or age-structured frameworks and improve model fidelity. Researchers are encouraged to develop multi-host models that explicitly include livestock, particularly cattle, to capture cross-species transmission dynamics and the human–animal interface. Such models should also account for environmental reservoirs, migratory wildlife, and seasonal fluctuations to better represent real-world transmission processes. Implementing ensemble modelling approaches across multiple frameworks can help quantify uncertainty, compare predictions, and improve confidence in outbreak forecasts, providing more reliable guidance for both short- and long-term planning.

Establishing standardized validation metrics, transparent reporting of model assumptions, and open-access parameter databases will facilitate reproducibility and enhance the credibility of model outputs. Without standardized reporting and rigorous uncertainty analysis, model outputs risk being misinterpreted by policy-makers, as the lack of comparability between studies can lead to conflicting guidance for outbreak preparedness. Policy-makers should critically assess model assumptions, validation results, and uncertainty estimates when using these models to guide decision-making. Reliance on single-model predictions should be avoided; instead, ensemble forecasts that synthesize outputs from multiple models can provide a more nuanced understanding of risk and potential outcomes. Simultaneously, investment in comprehensive surveillance and high-resolution data collection systems will generate timely information essential for informing and updating models. Funders can accelerate progress by supporting targeted data collection for parameterization, investing in the development of computationally intensive models such as agent-based and network models, and promoting transparency through requirements for open code, data sharing, and model validation studies. Together, these coordinated efforts across researchers, policy-makers, and funders will strengthen the ability of HPAI models to generate actionable insights, inform evidence-based outbreak preparedness, and guide effective interventions across wild birds, poultry farms, livestock farms, and human populations.

## Supporting information

S1 Appendix Literature search strategy. (PDF)

S1 Table Data extraction spreadsheet. (XLSX)

S2 Table PRISMA-ScR checklist. (PDF)
