# Supplementary material for: Mechanistic modelling of highly pathogenic avian influenza: A scoping review revealing critical gaps in cross-species transmission models
Source: PLoS One. 2026 Apr 30;21(4):e0347929. doi: 10.1371/journal.pone.0347929 (PMC13132250; doi:10.1371/journal.pone.0347929)
Supplement: S1 Appendix — (PDF) [file pone.0347929.s001.pdf]

Two databases were searched: PubMed and Web of Science. PubMed was selected for its comprehensive coverage of life sciences and biomedical literature, while Web of Science was included to broaden the scope and capture studies beyond biomedical research. The searches were conducted on 13 June 2025 using two complementary strategies to maximize retrieval.

PubMed:

- ((HPAI) OR (Avian influenza) OR (H5N1)) AND (model) AND ((mathematical) OR (math) OR (equation) OR (predictive) OR (surveillance) OR (forecast))
- Broadened Search Strategy:((HPAI) OR (Avian influenza) OR (H5)) AND (model) AND ((mathematical) OR (math) OR (equation) OR (predictive) OR (surveillance) OR (forecast))

Web of Science:

- ((ALL=(HPAI) OR ALL=(Avian influenza) OR ALL=(H5N1)) AND ALL=(model) AND (ALL=(forcast) OR ALL=(mathematical) OR ALL=(math) OR ALL=(equation) OR ALL=(surveillance) OR ALL=(predictive)))
- Broadened Search Strategy:((ALL=(HPAI) OR ALL=(Avian influenza) OR ALL=(H5)) AND ALL=(model) AND (ALL=(forecast) OR ALL=(mathematical) OR ALL=(math) OR ALL=(equation) OR ALL=(surveillance) OR ALL=(predictive)))
